# The effectiveness of mHealth mindfulness interventions on perinatal psychological health: a systematic review

**DOI:** 10.1093/oodh/oqaf006

**Published:** 2025-03-20

**Authors:** Prabhadini Godage, Oonagh M Giggins, Julie Doyle, Anita Byrne

**Affiliations:** NetwellCASALA, School of Health and Science, Dundalk Institute of Technology, Dublin Road Dundalk, County Louth A91 K584, Ireland; NetwellCASALA, School of Health and Science, Dundalk Institute of Technology, Dublin Road Dundalk, County Louth A91 K584, Ireland; NetwellCASALA, School of Health and Science, Dundalk Institute of Technology, Dublin Road Dundalk, County Louth A91 K584, Ireland; NetwellCASALA, School of Health and Science, Dundalk Institute of Technology, Dublin Road Dundalk, County Louth A91 K584, Ireland

**Keywords:** mHealth, mindfulness-based interventions, perinatal psychological health, depression, anxiety, narrative synthesis

## Abstract

Mindfulness-Based Interventions (MBIs) delivered via mobile health (mHealth) platforms have gained attention for improving perinatal psychological health. This review systematically examines the effectiveness of mHealth MBIs in improving perinatal psychological outcomes, including stress, anxiety and depression, with a secondary focus on safety, engagement, acceptability and dropout rates. A systematic search was conducted across PubMed, Cochrane Library, Science Direct, Scopus, ACM Digital Library and IEEE Xplore, along with grey literature, for English-language journal articles from inception until July 2024. All included studies were assessed for methodological quality using standardized critical appraisal instrument. Significant heterogeneity in study designs, program structures and data collection methods precluded meta-analysis, leading to a narrative synthesis of the results. Fifteen studies were included, featuring a mix of quantitative and qualitative designs. Findings indicated that mHealth MBIs demonstrated promise in reducing stress, anxiety and depressive symptoms during pregnancy and postpartum. However, participant engagement in these interventions was influenced by factors such as intervention complexity, user interface challenges and technological issues like app compatibility. Dropouts were commonly attributed to time constraints and technical difficulties, while user feedback emphasized the need for varied and flexible content to sustain interest and perceived effectiveness. Overall, mHealth MBIs offer potential benefits for perinatal psychological health, particularly in reducing stress and anxiety. However, maintaining high engagement and low dropout rates remains a challenge. Future studies should identify optimal intervention formats, enhance adherence and assess long-term impacts of mHealth MBIs to strengthen the evidence base, particularly in diverse settings and for pregnancy-related complications.

## INTRODUCTION

The perinatal period, encompassing both pregnancy and the first year postpartum, is a crucial period for the psychological well-being of women. In this period, perinatal individuals undergo significant physiological, hormonal and emotional transformations that may lead to increased vulnerability to mental health issues, especially stress, anxiety and depression [1]. Depression stands as the most prevalent perinatal mental health disorder, with global studies reporting perinatal depression rates of up to 20% both during pregnancy and the postpartum period [2–4]. Pregnancy-specific anxiety is also relatively common, impacting 12% to 20% of women [5]. Stress experienced during pregnancy is a major contributor to these psychological disorders. Pregnancy-related stress can come from different sources, such as physical discomfort, concerns about the health of the baby, changes in body image, financial and relationship strains and fear of childbirth [6]. Pregnancy-related complications like Gestational Diabetes Mellitus (GDM), pre-eclampsia and hyperemesis gravidarum, may worsen any pre-existing psychological concerns or trigger new ones [7]. Extended physiological stress response in expectant people can result in negative consequences such as elevated cortisol levels, preterm labour, low birth weight and complications during labour and delivery [8, 9]. Additionally, it can affect fetal neurodevelopment and may have long-term health implications for the child [10].

Given the significant influence of stress and other mental health disorders in the perinatal period, there is a pressing requirement for effective interventions that can help alleviate these conditions without jeopardizing the well-being of mother and baby. However, there are potential risks associated with administering psychotropic medication during pregnancy, including teratogenicity, preterm birth and long-term neurodevelopmental issues [11]. These concerns have caused an increasing interest in non-pharmacological treatments that can provide effective relief from psychological distress while minimizing the potential harm to the fetus [12].

Mindfulness-Based Interventions (MBIs) are becoming popular as a promising non-pharmacological approach to improving perinatal psychological health [13]. Mindfulness is defined as ‘self-regulated attention on present movement experience with an open, non-judgemental, and accepting attitude’ [14]. The theoretical foundation of mindfulness suggests that it can influence brain regions involved in emotional regulation, self-referential processing and stress response, making it particularly relevant in managing psychological challenges during pregnancy [15]. Studies have demonstrated that pregnant women who practice mindfulness techniques such as Mindfulness-Based Stress Reduction (MBSR) and Mindfulness-Based Cognitive Therapy (MBCT) experience a notable reduction in symptoms of depression, anxiety and stress, along with enhancement in general well-being [16, 17]. These interventions are especially appealing in the perinatal period due to their non-invasive nature and the absence of pharmacological risks. Nevertheless, traditional mindfulness programmes, which usually require attending in-person classes and group sessions, present several barriers to access due to scheduling conflicts, childcare needs, a lack of transportation and pregnancy discomfort, limiting participation in these programs.

In recent decades, the advent of digital health technologies has opened up new possibilities for delivering MBIs in a more accessible and flexible manner [18]. This was accelerated during the global COVID-19 pandemic, which saw the rise of remote healthcare solutions [19]. In this context, mobile health (mHealth) interventions have garnered attention for their potential to enhance treatment adherence and encourage disclosure of perinatal challenges [20]. mHealth interventions are delivered through smartphones and tablets, allowing users to engage in therapeutic activities at their own pace and from the comfort of their homes, which is especially beneficial for pregnant women who may face mobility issues or time constraints [21]. Moreover, mHealth platforms often include features such as notifications, progress tracking and personalized content, which can be crucial for effective mental health support [22].

Two existing systematic reviews have explored the impact of Digital Mindfulness-Based Interventions (DMBIs) on perinatal psychological health, demonstrating improvements in reducing anxiety, depression and stress during pregnancy and postpartum. Mefrouche et al. [23] conducted a meta-analysis demonstrating significant reductions in depressive and anxiety symptoms among pregnant populations engaging in digital mindfulness programs [23]. Similarly, Mao et al. [24] examined internet-delivered mindfulness programs, finding notable improvements in overall mental well-being and emotional resilience during the perinatal period [24]. However, these reviews focus more broadly on DMBIs and do not specifically address the unique advantages of mHealth MBIs, which leverage mobile technologies to provide more accessible, personalized and real-time support.

This review specifically focuses on mHealth MBIs, which offer distinct advantages over other digital formats. The portability and ease of use of mobile devices encourage user engagement and adherence, enabling pregnant individuals to integrate mindfulness practices into their daily routines. Additionally, the widespread availability of mobile phones, even in low-resource settings, ensures inclusivity and accessibility, allowing diverse populations to benefit from these interventions regardless of geographic or socioeconomic barriers [25]. Continuous access and timely support through mHealth make these solutions particularly valuable in prenatal care, where sustained psychological support is critical. By leveraging these unique capabilities, this systematic review will examine the effectiveness of mHealth MBIs in enhancing perinatal psychological health, addressing gaps in the current evidence base and offering insights into their potential for broader implementation.

Overall, this study aims to assess how effective mHealth MBIs are in addressing common challenges such as anxiety, depression and stress during pregnancy and postpartum. The review will also explore the safety, engagement, acceptability and dropout rates from mHealth MBIs in this cohort.

## METHODS

### Design and procedure

The present review was conducted following the Preferred Reporting Items for Systematic Review and Meta-Analysis (PRISMA) guidelines [26]. The review protocol was registered with PROSPERO, the International Prospective Register of Systematic Reviews, under the unique identifier CRD 42023489561. The review primarily focused on evaluating the effectiveness of mHealth MBIs in improving perinatal psychological health while also assessing their safety, engagement, acceptability and dropout rates as secondary outcomes.

### Eligibility criteria

This review targeted studies involving perinatal individuals aged 18 years or older, specifically pregnant women and/or those in the first year postpartum, with or without pregnancy-related complications such as GDM, Pregnancy Induced Hypertension (PIH) and heart diseases. The primary emphasis was on impact of mHealth MBIs with detailed assessments of outcomes related to perinatal psychological well-being, including stress, anxiety and depression. Included interventions encompassed structured mindfulness-based programs such as, MBSR, MBCT, Mindfulness-integrated Cognitive Behavioural Therapy (MiCBT) and other MBIs [27]. To maintain a clear focus on the effectiveness of mHealth MBIs for perinatal psychological health, digital web-based mindfulness interventions were excluded. MBIs solely delivered through messaging platforms like WhatsApp and WeChat, whether mobile or web-based, were also excluded. This was to ensure that the review captured the comprehensive and unique outcomes specifically tied to structured mHealth interventions, where mobile applications play a central role in delivering mindfulness practices, rather than relying on simple communication tools for interaction. Both quantitative and qualitative investigations, such as cohort studies, Randomized Controlled Trials (RCTs), non- RCTs, descriptive studies (e.g. surveys) and mixed method studies were included. Only studies published in peer-reviewed journals in the English language were included to enhance robustness of findings.

### Search strategy

A systematic search was conducted across multiple electronic databases, including PubMed, Cochrane Library, Science Direct, Scopus, ACM Digital Library and IEEE Xplore for English-language journal articles, without restrictions on the time period for the studies included, from inspection up to July 2024. Additionally, Google Scholar was also searched for any relevant gray literature, and the reference list of all selected articles was manually reviewed to ensure that any relevant studies not captured by the initial database searches were included. The search strategy was developed by combining four main subject heading domains (Mobile applications, Mindfulness, Mental health and Pregnancy) using the AND operator in alignment with the Sample, Phenomenon of Interest, Design, Evaluation and Research type (SPIDER) framework. These domains, along with relevant specific key terms aligned with each database's indexing system, were employed to conduct the search. Search strategies for the five selected databases are provided in Appendix 1.

### Screening and selection process

All search results were exported and managed using the Zotero software [28]. Duplicates were identified and removed within Zotero. First, titles and abstracts were screened by the first author to assess relevance based on the inclusion criteria. Full-text articles of studies meeting the criteria were retrieved for a detailed review. The decision to include or exclude an article was initially made by the first author and cross-checked by the second author. Any disagreement during the full-text review process was resolved through discussion and consensuses between authors. Uncertainty at this stage was clarified in consultation with the third author.

### Data extraction and synthesis

Data regarding study characteristics and outcome measures were extracted from the studies and recorded on two predesigned forms, with one dedicated to primary data and another for secondary data. The primary data table captured study characteristics, intervention types, key psychological outcomes (stress, anxiety, depression) and significant findings related to mHealth MBIs. The secondary data extraction focused on safety, participant engagement, acceptability and dropout rates. The first author initially inputted the data, which was subsequently cross-checked against the original studies by the second author to ensure accuracy. Any discrepancy was resolved through discussions and consensus, with input from a third author when necessary.

Due to the wide variation in study methodologies, the structure of mindfulness programs, participant samples, data collection points and the instruments used to collect the data, it was deemed inappropriate to perform a meta-analysis. Instead, a narrative synthesis was conducted to comprehensively assess the findings. This approach allowed for a detailed exploration of the evidence, highlighting trends and differences across the studies, without relying on statistical aggregation.

### Assessment of risk of bias and quality

Eligible studies were assessed for risk of bias and methodological quality by the first and second authors independently using the Mixed Method Appraisal Tool (MMAT) [29]. The MMAT was chosen as it is specifically designed to appraise a wide range of study designs, which aligns with the diverse methodologies included in this review. The risk of bias in each study was categorized as low, moderate or high. Any disagreement in the quality assessment was resolved through discussion until a consensus was reached.

### Ethical considerations

As this study involved secondary data collection through a review of published literature, no direct ethical approval was required. However, ethical considerations were upheld by ensuring that all included studies had obtained relevant ethical approvals, where applicable, and followed appropriate guidelines for participant consent, privacy and data protection. This review also adhered to the principles of transparency and integrity in research reporting.

## RESULTS

### Overview of study selection

**Figure 1:**
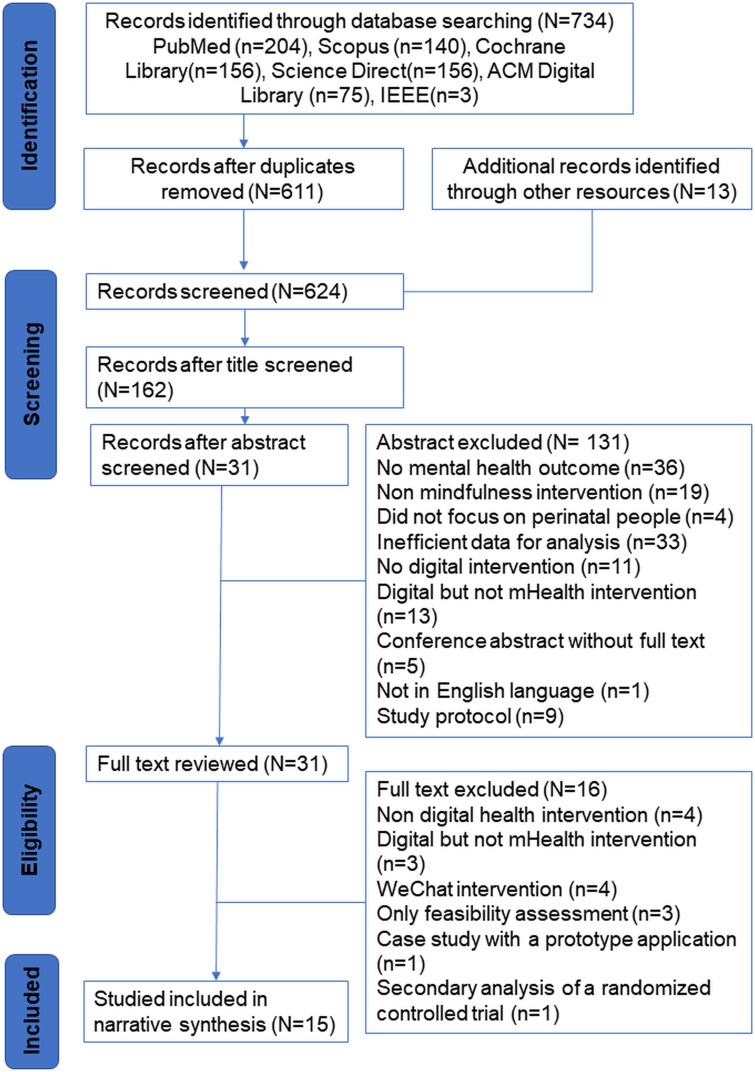
Prisma flow chart.

The search yielded 734 articles with 123 duplicates removed. Thirteen additional records were identified through other resources. After the title screening, 462 articles were removed, and an additional 131 were excluded following the abstract review. Following full-text review, a total of 16 studies were excluded for reasons outlined in Fig. 1. The exclusions included studies that focused on non-digital health interventions, digital but not mHealth interventions or used platforms like WeChat, which involved web and mobile-based tools but did not align with the specific mHealth focus of this review. Additionally, studies that only assessed feasibility, involved case studies with prototype applications or presented secondary analyses of previously included trials were excluded due to insufficient data or lack of relevance to the effectiveness of mHealth MBIs. Finally, 15 articles were included in the narrative synthesis of this review.

### Characteristics of included studies

The majority of studies were conducted in the United States (46.7%, 7/15) [22, 30–35], followed by China (20%, 3/15) [36–38] and Germany (13.3%, 2/15) [39, 40]. The remaining studies were conducted in Italy (6.7%, 1/15) [41], India (6.7%, 1/15) [42] and New Zealand (6.7%, 1/15) [43]. 93% (14 out of 15) of the studies were conducted within the past five years.

Among the 15 included studies, seven were RCTs, encompassing various RCT designs (e.g. parallel-group, multi-site, pre-to-post RCT design). Six studies used non-RCTs, including two pilot studies, one quasi-experimental design, one longitudinal panel study, one single-arm trial and one prospective cohort study. Additionally, two studies employed a mixed-methods design. Participants across all studies were women aged 18 years or older, categorized into four health conditions at baseline: women with mental health challenges (33.3%, 5/15), healthy perinatal women (46.6%, 7/15), hospitalized pregnant women (13.3%, 2/15), and one study focused on women with obesity-related risks (6.7%, 1/15). In terms of the perinatal period studied, 13 studies were conducted during pregnancy, with six of those also collecting data into the postnatal period. Two studies focused exclusively on postpartum women, one recruiting participants up to 6 months postpartum and the other involving mothers of children aged 0–12 months [30, 43]. Sample sizes varied widely, ranging from five to 512 participants. Six studies included more than 100 participants, while six studies had sample sizes between 27 and 84, and four had fewer than 50 participants. Detailed characteristics of the included studies are listed in Table 1.

**Table 1:** Characteristics of included studies (source: Author).

**Authors & Year**	**Country**	**Design**	**Participants**	**Nature of mindfulness intervention**	**Primary and other outcomes measures**	**Significant between-group differences**	**Significant within-group differences**
Balsam *et al*., 2023 [31]	USA	Longitudinal, single-arm trial	Pregnant women	• 1-month mindfulness (N = 20); • Headspace app: 'Basics' + 'Pregnancy' courses (60 meditations, 530–1050 minutes total).	Anxiety: GAD-7 Pregnancy Anxiety: PRAS Stress: PSS		• ↓Anxiety and stress • ↓Low-frequency heart rate variability.
Sun *et al*., 2021 [38]	China	Single-center, two-parallel-armed, assessor-blinded, 1:1-allocated RCT	Pregnant women and postpartum women	• 8-week MBCT via Spirits Healing app - N = 84 I (Intervention); • Treat as usual N = 84 C (Control). Daily formal and informal mindfulness practices	Depression - EPDS Anxiety - PHQ-9GAD-7 Perceived stress - PSS Positive affect - PA Negative affect - NA Sleep-related problems -PSQI Fatigue - FSS Prospective memory -PM Retrospective memory - RM Fear - WDEQ	• ↓Depression, anxiety, positive affect and stress • ↓ Risk of depressive symptoms and higher rates of depression symptom remission post-intervention	• EPDS score in the mindfulness training group continued to decline at 8 weeks, remained at a low level at 18 weeks and increased slightly postpartum
Doty *et al*., 2022 [32]	USA	Multi-site randomized trial	Pregnant women	• 4-day mindful meditation with Calm app (N = 28 I); • Treat as usual (N = 28 C)	Maternal state anxiety- STAI Stress - PSS) Depression - EPDS	• ↓ Anxiety. • Stress and Depression scores on day 4 were similar across both groups	• Intervention Group (IG) - larger decrease in STAI scores from day 1 to day 4.
Avalos *et al*., 2020 [30]	USA	Mixed-methods single-arm trial	Postpartum women	• 6-week mindfulness meditation with Headspace app (N = 27); • Daily 10–20 minute sessions starting with the 30-day basics course	Depression - PHQ-8 Stress - 10-item PSS Sleep quality - 19-item PSQI Mindfulness - 24-item FFMQ		IG -↓ Depressive symptoms and perceived stress, ↑Sleep quality and mindfulness
Goetz *et al*., 2020 [39]	Germany	Pilot Study	Pregnant women	• 1-week mindfulness-based CBT with MindMom app (N = 68); • Three 45-minute modules: mindful breathing (day 1), body scan (day 3) and "loving kindness" (day 5)	Depression - EPDS Anxiety symptoms - STAI Mindfulness - PRAQ-R.		• ↓State anxiety levels significantly reduced. • ↓Pregnancy-related anxiety scores in the second assessment. • No significant changes in depression scores
Hassdenteufel *et al*., 2023 [40]	Germany	A multicenter, randomized controlled study (prospective study)	Pregnant women and postpartum women	• 8-week mindfulness-based CBT via eMBI app (N = 230); • Supervised weekly sessions (45 min) from weeks 29 to 36 of pregnancy, with follow-up up to 5 months postpartum. • Treat as usual (N = 230)	Depression - EPDS Anxiety - STAI Pregnancy-related anxiety - PRAQ-R Mindfulness - FMI-14/FFA-14. Mental disorders for primary care - PHQ-D	• No significant interaction effects - depressive symptoms and anxiety. • IG - six weeks postpartum - • ↓Risk for adverse mental outcomes and ↓pregnancy and birth-related anxiety	• IG - Mindfulness scores improved significantly
Liu *et al*., 2022 [37]	China	Randomized controlled trial	Pregnant women and postpartum women	• 8-week We’ll App mindfulness program (N = 65); • Daily meditation and support activities (20-minute webinar, 3 times a week). • Treat as usual (N = 65).	Depression - EPDS Parenting self-efficacy - PMPS Perceived social support - MSPSS Mindfulness - MAAS	• ↑Perceived social support and maternal self-efficacy • ↓Postpartum depressive symptoms • Mindfulness - no significant effect	• Control Group (CG), no significant differences - four variables between pre-test and post-test
Bear *et al*., 2022 [43]	New Zealand	The study used a pre- to post-RCT design	Mothers of children aged 0–12 months	• 8-week Smiling Mind App Mindfulness Foundations program (N = 49 I); • 10 modules with daily sessions (1–43 minutes each, avg. 9.20 min); • Baby +Tracker Application (N = 50 C)	Levels of distress - DASS21 Mindful attention - MAAS	• ↓Depression, anxiety and stress and ↑mindful attention	
Carissoli *et al*., 2022 [41]	Italy	Quasi-experimental controlled design	Pregnant women and postpartum women	• 4-week BenEssere Mamma app program (N = 57); • 20 daily meditation exercises focusing on stress, emotional awareness and experiential activities; • Treat as usual (N = 51).	Level of psychological well-being - Ryff's Psychological Well-being Scale.	• ↑Sense of autonomy and self-acceptance after childbirth	
Ward *et al*., 2023 [35]	USA	Pilot Study	Pregnant women	• 30-day Headspace app (N = 5); • Guided mindfulness meditation focused on pregnancy well-being and mindful eating (10 minutes daily, with an optional additional 10 minutes);	Depression - EPDS Anxiety - PRAQ Perceived stress - PSS Maternal affect - PANAS, MEQ,TFEQ.		• Decrease in negative affect • ↓Perceived stress was observed exclusively in the pregnancy plus mindful eating module group. • Participants in the mindful eating module - ↑MEQ scores, in pregnancy modul - no change.
Kubo *et al*., 2021 [22]	USA	Single-arm trial	Pregnant women	• 6-week Headspace app (N = 27); • 30-day "Basics" course followed by themed courses on pregnancy, anxiety, relationships and sleep (10–20 minutes daily);	Depression - 8-item PHQ-8 Stress - 10 item PSS • Sleep Quality - 19-item PSQI Mindfulness - 24-item FFMQ-SF		• improved Depression symptoms, perceived stress, sleep quality and mindfulness
Smith *et al*., 2021 [34]	USA	Randomized controlled trial	Pregnant women	30-day Calm app usage N = 50 (I); Participants encouraged to use any app component (mindfulness meditation, sleep stories, nature sounds) for 70 minutes weekly (10 minutes daily); Treat as usual N = 51 (C)	Self-reported perceived stress - Cohen’s 10-item PSS. Depression, anxiety and sleep disturbance - Zigmond’s 14-item Hospital Anxiety and Depression Scale and the 8-item PROMIS and Sleep Disturbance Short Form.	• ↓ Perceived stress and self-reported depression and anxiety, • ↓ Sleep disturbance	
Porter *et al*., 2022 [33]	USA	Prospective Cohort study	Pregnant women	Mindfulness meditation via Expectful app N = 21 (I); Daily meditations tailored to trimester and physical/emotional states (10–20 minutes each); Treat as usual N = 38 (C)	Stress - PSS	• ↓PSS scores	
Leng *et al*., 2023 [36]	China	Parallel-group, randomized controlled trial	Pregnant women and postpartum women	Mindfulness-based CBT via "Thriving in Pregnancy" app + WeChat N = 38(I); Eight weekly sessions with tailored mindfulness practices (20–40 minutes daily); web-based perinatal education programme N = 37 (C). Therapist assistance +self-guided	Depression - EPDS Perceived stress - PSS Trait mindfulness -FFMQ Positive affect - PA Self-compassion - SCS	• ↑Depression scores, Trait mindfulness, Self-compassion, Stress reduction, Positive affect, • ↓Emergent cesarean section, • ↑Higher Apgar scores in infants.	• IG - trait mindfulness, self-compassion and positive affect after the intervention either remained unchanged or decreased at 4–6 weeks postpartum.
Patil and Malhotra, 2021 [42]	India	longitudinal study (panel study) (Mixed method)	Pregnant women and postpartum women	Daily guided mindfulness meditation (N = 255, I); 8 minutes each day; Treat as usual (N = 257, C).	Responses on psychological scales: PSQI, PSRS and Cranley’s MFA scale	• ↑Sleep, • ↓Stress levels, fears related to childbirth, and postpartum blues. • ↑Cranley’s MFA scale score.	• IG - PSRS score is very low in 35 weeks compared to 15 weeks.

(GAD-7: Generalized Anxiety Disorder-7, PRAS: Pregnancy-Related Anxiety Scale, PSS: Perceived Stress Scale, EPDS: Edinburgh Postnatal Depression Scale, PHQ-9: Patient Health Questionnaire-9, PA: Positive Affect, PANAS: Positive and Negative Affect Schedule, NA: Negative Affect, PSQI: Pittsburgh Sleep Quality Index, FSS: Fatigue Severity Scale, PM: Prospective and Retrospective Memory Questionnaire, RM: Prospective and Retrospective Memory Questionnaire, WDEQ: Wijma Delivery Expectancy Questionnaire, STAI: State–Trait Anxiety Inventory, PHQ-8: Patient Health Questionnaire-8, FFMQ: Five Facet Mindfulness Questionnaire, PRAQ-R: Pregnancy-Related Anxiety Questionnaire – Revised, FMI-14/FFA-14: Freiburg Mindfulness Inventory, PMPS: Perceived Maternal Parental Self-Efficacy tool, MSPSS: Multidimensional Perceived Social Support Scale, MAAS: Mindful Awareness Scale, DASS21: Depression Anxiety and Stress Scale Short Form, MEQ: Mindful Eating Questionnaire, TFEQ: Three-Factor Eating Questionnaire, PROMIS: Reported Outcomes Measurement Information System, SCS: Self-Compassion Scale, RESE: Emotional Self-Efficacy, MT: Monitoring Training, MAT: Monitoring with an Emphasis on Acceptance Training, PSRS: Perceived Stress Reactivity Scale, PSQI: Pittsburgh Sleep Quality Index Scale, CMFAS: Cranley’s Maternal–Fetal Attachment Scale.)

### Characteristics of mHealth mindfulness studies

The MBIs delivered through mHealth platforms in the included studies primarily focused on guided mindfulness practices. The majority of interventions were purely mindfulness-based (73.3%, 11/15), while the rest integrated mindfulness with other components such as Cognitive Behavioural Therapy (CBT) or psychoeducation (26.6%, 4/15). All studies used mobile applications as the primary method of delivery. However, in one study [44], the app also incorporated self-learning modules embedded within WeChat. All interventions were self-guided (93.3%, 14/15), except for one study [36] that incorporated therapist support through WeChat. The intervention periods varied, with most being delivered during pregnancy (86.7%, 13/15), while two studies targeted individuals during the postpartum period. The MBIs duration ranged from four days to eight weeks in most studies, with four interventions lasting four weeks and five interventions extending to eight weeks. The frequency of mindfulness practice ranged from three times a week to twice daily, with session lengths varying from as short as three minutes to as long as 45 minutes. Evaluation time points across studies also varied considerably, with several studies assessing outcomes at multiple time points, including follow-up periods extending from four weeks postpartum to as long as five months postpartum.

### Evaluation of primary and secondary outcomes in mHealth MBIs

The primary outcomes in the included mHealth mindfulness intervention studies were predominantly focused on mental health symptoms such as anxiety, depression and stress. A majority of studies used validated scales to assess outcomes. For instance, anxiety and pregnancy-related anxiety were commonly measured using instruments like the GAD-7 (Generalized Anxiety Disorder – 7 Item Scale) and PRAS (Pregnancy-Related Anxiety Scale) [31], while depression was frequently evaluated with the EPDS (Edinburgh Postnatal Depression Scale) [37, 38]. Stress was another frequent measure, assessed through the PSS (Perceived Stress Scale) in various studies [30, 32, 34]. Sleep quality, mindfulness and psychological well-being were also central measures in several studies [22, 30, 41]. Other secondary outcomes varied across studies, reflecting the diverse nature of mHealth MBIs. These outcomes included physiological metrics like heart rate variability [31], parenting self-efficacy [37] and acceptance and feasibility of the interventions [34, 35, 39]. Some studies also explored obstetric and neonatal outcomes, including birth weight, delivery type and neonatal Apgar scores [36, 42].

### Impact of mHealth MBIs on perinatal psychological health

Depression was reported in eleven studies, comprising seven RCTs and four non-RCTs. Among the RCTs, five studies found significant reductions in depression symptoms post-intervention, with effects greater than those observed in control groups [34, 36–38, 43]. However, two studies did not report significant changes in depression scores [32, 40]. In the non-RCTs, three studies demonstrated significant reductions in depression, indicating potential improvements within the intervention groups over time [22, 30, 35]. One study did not observe any significant differences in depression outcomes [39].

Anxiety was assessed in eight studies, including five RCTs and three non-RCTs. Of the RCTs, three [34, 38, 43] demonstrated significant differences between the intervention and control groups, with the intervention groups showing notable improvements in anxiety levels. In contrast, two RCTs [32, 40] reported improvements within the intervention groups but found no significant differences when compared to controls. Among the non-RCTs, two studies [31, 35] highlighted marked reductions in anxiety within the intervention groups, particularly in pregnancy-related anxiety, while one study [39] observed only slight improvements.

Stress was another frequently reported outcome, with four RCTs demonstrating significant between-group differences, underscoring the effectiveness of MBIs in reducing perceived stress [34, 36, 38, 43]. However, one RCT did not observe significant between-group changes in stress levels post-intervention [32]. In the non-RCTs, six studies [22, 30, 31, 33, 35, 42] reported significant within-group reductions in stress, indicating improvements over time.

Mindfulness was measured in six studies, including four RCTs and two non-RCTs. Among the RCTs, three studies reported significant improvements in mindfulness following the intervention [36, 40, 43], while one study found no significant difference between groups [37]. In the non-RCTs, both studies [22, 30] showed significant improvements in mindfulness within the intervention groups.

Physiological and behavioural measures such as sleep quality and heart rate variability were assessed in some studies, reporting reductions in physiological stress markers [31] and observing significant within-group improvements in sleep quality [30]. However, sleep outcomes were less frequently reported in all RCTs.

### Secondary outcomes: safety, engagement, acceptability and dropout rates

Secondary data were extracted to provide a more comprehensive evaluation of factors that influence the overall effectiveness and feasibility of mHealth MBIs. Of the studies reviewed, four reported on all four secondary outcomes assessed in this review: safety, engagement, acceptability and dropout/retention. Nine studies reported at least two or three of these outcomes, while two studies did not report any secondary outcomes.

The safety of mHealth MBIs appears to be generally well-supported, though explicit reporting on safety was limited across the studies. Some studies reported no serious adverse events, indicating a low risk associated with these interventions [34, 40]. While one study [38] implemented safety measures by reminding participants that the app was not a substitute for psychotherapy, and encouraging professional medical support when necessary, another study [36] reported a miscarriage in one participant, though the event could not be definitively linked to the intervention.

Engagement levels varied widely among the studies. Completion rates ranged from 57% to 96.4%. For example, one study reported 55.6% completion, with 33.3% of participants completing all follow-ups [38]. The average adherence to intervention sessions ranged from 23% to 77% of the planned days.

Acceptability was generally high across the studies, with most participants reporting positive experiences and satisfaction with the interventions. Satisfaction rates ranged from 67% to 89.5%, with participants appreciating the convenience and perceived benefits of the mindfulness apps. Participants in two studies [22, 30] reported high satisfaction with the Headspace app, citing its ease of use, variety of meditation options and overall convenience. In contrast, one study [35] noted negative feedback from one participant, who found the mindful eating module overly focused, which diminished its effectiveness by negatively impacting her appetite.

Dropout rates showed considerable variability, reflecting different levels of participant retention. Post-intervention dropout rates ranged from 8.2% to 61%, with a substantial number of studies reporting rates between 30% and 50%. Follow-up retention rates also varied, with some studies achieving high retention (e.g. 100% at follow-up) while others experienced significant dropout (e.g. 43% and 57% dropout rates at follow-up). These differences highlight the challenge of maintaining participant engagement over time. Reasons for dropout included time constraints, with participants struggling to incorporate app usage into their daily lives due to family responsibilities, work and other prenatal obligations and technical issues such as phone compatibility and difficulties navigating app features [43]. Additionally, hospital-related factors such as early discharge, premature birth or Leave Against Medical Advice (LAMA) disrupted participation and psychological challenges, including elevated stress, anxiety and adverse pregnancy complications, further hindered retention in interventions conducted in hospital setups [32, 39]. Some participants, despite complying with meditation protocols, declined to complete post-intervention surveys, suggesting survey fatigue or perceived irrelevance of follow-up assessments as potential factors. [32].

Table 2 outlines the secondary data extraction information from the included studies.

**Table 2:** Secondary outcomes of studies (safety, Engagement, Acceptability and dropout) (source: Author).

**Authors & Year**	**Safety Evidence (Adverse Events and Safety Measures in the Study)**	**Engagement (Engagement Frequency and Duration in the Interventions)**	**Acceptability (User Satisfaction and Qualitative Feedback)**	**Dropout Rates (Retention Rates and Dropout Reasons)**
Balsam *et al*., 2023 [31]		13/20 completed 65% with ten of them achieving95% or higher.	↑Adherence	100% participant retention 100% retention at follow-up assessments
Sun *et al*., 2021 [38]	Reminded app is not a substitute for psychotherapy and encouraged to seek professional support if needed.	55.6% completed at least three follow-ups 33.3% completed all follow-ups		IG - 29% - dropped out CG - 41% of dropped out Overall dropout - 34.5%
Doty *et al*., 2022 [32]		96.4% completed at least one meditation session 39.3% completed all	CG - 88.8% reported a positive experience IG - 89.5% reported a positive experience	Retention at post-intervention: 72.7% Retention at last follow-up: 71.4%
Avalos *et al*., 2020 [30]		Initial App Usage: 74% - at least once. Usage Among Completers**:** 100% - at least once. 26% practiced 70% of the days. Continued Usage: 58% continued at least once during the following month	69% expressed high satisfaction. Intent to Continue: All participants User Experience: Easy to use, valued variety and convenience.	Retention at post-intervention - 70% Retention at last follow up - 70%
Goetz *et al*., 2020 [39]		Completion rate: 57%		Dropout rate: 43%
Hassdenteufel *et al*., 2023 [40]	No serious adverse events were recorded.			38.26% drop out, in intervention 25.35% drop out, in control
Liu *et al*., 2022 [37]				
Bear *et al*., 2022 [43]		Post-intervention (8 weeks) IG: 90% & CG: 86% engaged in 3+ sessions/week. IG: 6% & CG: 4% engaged in 1–2 sessions/week. IG: 4% & CG: 10% engaged in <1 session/week. 4 week follow-up Both groups: 93% engaged in 3+ sessions/week. IG: 7% & CG: 0% engaged in 1–2 sessions/week. IG: 0% & CG: 7% engaged in <1 session/week.		Retention rates: 43% at (8 weeks) 4-90% at (4 week follow-up)
Carissoli *et al*., 2022 [41]		86.1%) submitted practice reports 9 exercises over the four-week period	Rated the app as moderately pleasant and engaging. Preferences for app use: 36.4% Preferred weekly use, 30.3% Sometimes, and 15.2% as needed.	68.5% completed the pre and post-intervention questionnaires 39.1% completed the postpartum follow-up evaluation
‘Ward *et al*., 2023 [35]	One found mindful eating module negatively impacted appetite..	Exclusively pregnancy module users - 100% Common pregnancy module users - 77% Pregnancy and mindful eating modules users - 67%	All reported experiencing some level of perceived benefit None reported any negative experiences.	Retention at post-intervention (%) - 100% Retention at last follow up - 100%
Kubo *et al*., 2021 [22]		95% used Headspace at least once 58% practiced on ≥50% of days during 6 weeks. 26% practiced on ≥70% of days. 53% used post-intervention (1/month)	67% expressed high satisfaction (user-friendly and convenient)	74% completed baseline and follow-up surveys
Smith *et al*., 2021 [34]	No reported an adverse event or unintended effect.	37% - used the app ≥5/weeks 37% used a 3–4/per week	81% rated "very satisfied" or "satisfied" 86% found it easy or very easy to use.	Dropout after baseline assessments: 13.4% (IG), 7.7% (CG). Dropout before mid-study assessments: 36.5% (IG), 23.1% (CG).
Porter *et al*., 2022 [33]			84% recruitment rate shows strong interest	Retention at post-intervention (%) - 90% Retention at last follow up - 57.%
Leng *et al*., 2023 [36]	On adverse event—a miscarriage in the second trimester.	92.2% engaged in discussions at least six times		Retention at post-intervention - 92.2% Retention at 37 weeks - 76.3% Retention at last follow up - 86.8%
Patil and Malhotra, 2021 [42]				

### Quality of included studies

The quality assessment of the 15 studies included in this systematic review, based on the MMAT, reveals considerable variability in methodological rigor. Studies that scored 3/5 included two non-RCTs, reflecting moderate quality in design and execution [31, 39]. Conversely, two RCTs scored 1/5, suggesting significant limitations in their study design or reporting [32, 43]. Mixed methods studies also varied in quality, with scores of 2/5 [30] and 1/5 [42], highlighting challenges in integrating quantitative and qualitative data. The MMAT results are depicted in Table 3 below.

**Table 3:** MMAT quality assessment results (source: Author).

	**Studies**												**Criteria from the Mixed Methods Appraisal Tool**																	
		**1.1**	**1.2**	**1.3**	**1.4**	**1.5**	**2.1**	**2.2**	**2.3**	**2.4**	**2.5**	**3.1**	**3.2**	**3.3**	**3.4**	**3.5**	**4.1**	**4.2**	**4.3**	**4.4**	**4.5**	**5.1**	**5.2**	**5.3**	**5.4**	**5.5**	**Total Score**	**Overall Quality**		
		**Qualitative**					**Quantitative randomized controlled trials**					**Quantitative non-randomized**					**Quantitative descriptive**					**Mixed Methods**								
**1**	Balsam *et al*., 2023											**1**	**1**	**1**	**0**	**0**											**3/5**	**Moderate**		
**2**	Sun *et al* 2021						**1**	**1**	**0**	**1**	**0**																**3/5**	**Moderate**		
**3**	Doty *et al* 2022						**1**	**0**	**0**	**0**	**0**																**1/5**	**Low**		
**4**	Avalos *et al* 2020																					**1**	**0**	**0**	**1**	**0**	**2/5**	**Low**		
**5**	Goetz *et al* 2020											**1**	**1**	**0**	**0**	**1**											**3/5**	**Moderate**		
**6**	Hassdenteufel *et al* 2023						**1**	**0**	**1**	**0**	**0**																**2/5**	**Low**		
**7**	Liu *et al* 2022						**0**	**1**	**1**	**0**	**0**																**2/5**	**Low**		
**8**	Bear *et al* 2022						**0**	**1**	**0**	**0**	**0**																**1/5**	**Low**		
**9**	Carissoli *et al* 2021											**0**	**1**	**0**	**0**	**0**											**1/5**	**Low**		
**10**	Ward *et al* 2023											**0**	**1**	**1**	**0**	**0**											**2/5**	**Low**		
**11**	Kubo *et al* 2021											**1**	**1**	**1**	**0**	**0**											**3/5**	**Moderate**		
**12**	Smith *et al* 2021						**1**	**1**	**0**	**0**	**0**																**2/5**	**Low**		
**13**	Porter *et al* 2022											**1**	**1**	**0**	**0**	**0**											**2/5**	**Low**		
**14**	Leng *et al* 2023						**1**	**0**	**1**	**0**	**1**																**3/5**	**Moderate**		
**15**	Patil and Malhotra 2021																					**1**	**0**	**0**	**0**	**0**	**1/5**	**Low**		

## DISCUSSION

This review comprehensively synthesized the available evidence to present the characteristics of mHealth MBIs and explored their effectiveness in improving perinatal psychological health.

### Interpreting the findings on the effectiveness of mHealth MBIs on perinatal psychological health

This review cannot draw definitive conclusions about the overall effectiveness of mHealth MBIs in perinatal psychological health due to the small number of studies, varying study quality and differences in study designs. However, the evidence suggests that mHealth MBIs may significantly reduce maternal depressive symptoms, alleviate anxiety and stress and improve mindfulness, sleep quality and self-compassion, although the consistency of these effects varied across the studies.

Previous reviews of MBIs for perinatal women have demonstrated the potential of MBIs in alleviating mental health symptoms, such as depression and anxiety [44, 45]. However, the specific advantages of using mHealth technology-based mindfulness interventions over traditional face-to-face and other web-based digital interventions remain underexplored. Compared to face-to-face support, mHealth interventions provide unparalleled flexibility, allowing women to access and engage with the content on their own schedule, which is particularly useful during pregnancy and postpartum when mobility and time are often limited [46]. Additionally, mHealth MBIs remove barriers related to location, making psychological support more accessible to those in rural or underserved areas where healthcare services may be scarce [47].

When compared to web-based DMBIs, mHealth MBIs leverage the high mobile phone penetration, offering ease of use through apps that can be accessed anywhere, anytime, without needing a computer or stationary setup [48]. This portability ensures that pregnant and postpartum women can engage in brief, frequent practices throughout their daily routines. mHealth interventions also often include features like reminders and progress tracking, which can enhance adherence and personalization. These advantages make mHealth a uniquely convenient and scalable solution for delivering psychological support during the perinatal period, particularly in settings with limited healthcare infrastructure.

Although this review provides early evidence supporting the effectiveness of mHealth MBIs, particularly in reducing depressive symptoms, the limited quality of some studies raises concerns. Most studies lacked long-term follow-up and did not assess the clinical significance of the improvements in depression and anxiety. For example, while many reported reductions in symptoms, none of the studies measured whether these changes were clinically meaningful, using tools such as the EPDS. Future research should focus on determining the Minimal Clinically Important Difference (MCID) to establish the true impact of these interventions [49, 50].

Regarding mechanisms of action, relatively few studies have examined factors such as mindfulness and self-compassion as mediators of the psychological improvements seen with mHealth MBIs for perinatal women. For instance, an increase in mindfulness was identified as being linked to better emotional well-being [22]. However, research remains sparse on the underlying processes driving these benefits, especially for the perinatal population. Furthermore, only a small number of studies have assessed secondary outcomes like maternal anxiety related to childbirth or biological stress markers, which are critical indicators of maternal mental health.

In conclusion, while early findings indicate that mHealth MBIs may improve perinatal psychological health by reducing depressive symptoms and fostering mindfulness, the overall results remain inconclusive due to variability in the quality and quantity of the available studies. Future research should focus on addressing these gaps by exploring the clinical significance of these improvements, comparing the effectiveness of mHealth MBIs with both face-to-face and other digital interventions and further investigating the mechanisms underlying their success in perinatal care.

### Insight on safety, engagement, acceptability and retention

Safety reporting across the reviewed studies was generally sparse but indicated no major concerns. A few studies explicitly addressed safety [34, 40], which noted no serious adverse events. This aligns with findings from other mHealth intervention reviews where adverse events were rare, reflecting the generally low-risk nature of such interventions [51].

In terms of engagement, there was significant variability in participant adherence. Completion rates across studies ranged from as low as 57% to as high as 96.4%. This is comparable to other mHealth psychological interventions where participant engagement tends to fluctuate based on intervention complexity and user interface [52]. For instance, one study [43] reported technological difficulties such as app compatibility, which could contribute to lower engagement, mirroring similar challenges seen in other mHealth programs [53]. Furthermore, studies with structured reminders or additional support [35] tended to show higher adherence, suggesting that personalizing reminders or incorporating support mechanisms may help to improve engagement [54]. This variability suggests that while some interventions achieved high participant engagement, others faced challenges in maintaining consistent participation.

Acceptability was generally high across most studies, with positive participant feedback about the convenience and utility of mHealth MBIs. For example, some studies [32, 34] reported satisfaction rates exceeding 85%, a level consistent with other studies on digital mental health interventions for perinatal women [55]. However, negative feedback was also noted, with one participant finding a specific mindfulness module overly focused, demonstrating the importance of content variability in maintaining user interest and perceived effectiveness [35]. These findings indicate that while mHealth MBIs are well-accepted, customizing content and providing flexible usage options can further enhance user experience.

Dropout rates were notably high, reflecting a significant retention challenge. Only four studies (26.7%) reported dropout rates below 25%, while others experienced dropout rates ranging from 30% to 50%. These dropout rates were higher than those reported in traditional, face-to-face MBIs, which typically see dropout rates around 20% [38]. Reasons for dropout varied, including time constraints, technical issues, hospital-related factors such as early discharge or premature birth and psychological challenges like elevated stress and anxiety. Retention rates at follow-up also varied widely, with some studies achieving 100% retention, while others reported significant dropouts [39]. These findings underscore the multifaceted nature of dropout in MBIs, requiring flexible designs and targeted support to address these barriers.

Overall, the findings illustrate a wide range of outcomes in terms of safety, engagement, acceptability and retention for mHealth MBIs, indicating that while these interventions can be effective and well-received, maintaining high engagement and low dropout rates remains a challenge across different study settings.

### Limitations in previous mHealth MBIs

Several limitations were evident across the reviewed studies. First, the majority of studies included in this review were conducted in the United States (46.7%, 7/15), followed by China (20%, 3/15), Germany (13.3%, 2/15) and other countries such as Italy, India and New Zealand (each 6.7%, 1/15). This geographical skew suggests that mHealth MBIs for perinatal women are still in their infancy and predominantly evaluated in high-income countries, with limited representation from developing nations. More studies in resource-constrained settings are necessary to evaluate the accessibility and effectiveness of mHealth MBIs in contexts with fewer mental health resources.

Second, there was a notable variation in sample sizes. While six studies included more than 100 participants, the remaining studies had smaller sample sizes, with six studies ranging from 27 to 84 participants, and four studies enrolling fewer than 50 participants. This disparity in sample size affects the statistical power and generalizability of the findings. Additionally, the diversity in intervention designs further complicates the comparability of results, with seven studies using RCTs and six employing non-RCTs, such as quasi-experimental, longitudinal panel studies and single-arm trials. The use of non-RCT designs decreases the reliability of findings due to potential bias, especially when combined with the small sample sizes.

Third, the duration of interventions varied, with some lasting as short as four days and others extending up to eight weeks. The frequency and length of mindfulness practices also varied, ranging from short three-minute sessions to longer 45-minute sessions, delivered three times a week to twice daily. This inconsistency in intervention length and frequency may impact the comparability of outcomes, as longer and more frequent interventions typically yield more robust effects on psychological outcomes.

Moreover, only 53.3% of studies (eight studies) reported follow-up data, making it difficult to assess the long-term effectiveness of mHealth MBIs for perinatal individuals. Examining long-term outcomes would provide valuable insight into the sustainability of the benefits observed post-intervention and could inform the ideal timing for follow-up interventions.

Finally, the risk of bias was high across several studies, primarily due to deviations from planned interventions and reliance on self-reported outcomes, which is a common limitation in internet-delivered interventions. Non-blinded research designs further contribute to potential biases, leading to inaccurate assessments. For instance, the lack of blinding in non-RCTs may have influenced participant perceptions, thereby inflating self-reported improvements in mental health outcomes.

In summary, despite the promising potential of mHealth MBIs in perinatal care, the field is still emerging. Future research should prioritize rigorous study designs, larger sample sizes and follow-up assessments, while also expanding geographic representation to ensure the interventions are relevant and effective across diverse populations.

### Diversity in mHealth MBIs approaches

The mHealth MBIs reviewed varied significantly in terms of delivery formats, intervention components and the duration and intensity of sessions. This diversity reflects the flexibility of mobile platforms in accommodating different user preferences and needs. Some interventions were self-guided, while others incorporated varying degrees of professional guidance. Both types showed potential effectiveness, but the optimal approach remains unclear. Previous studies have highlighted the benefits of therapist-guided interventions, yet self-guided programs are more accessible and cost-efficient [56, 57]. However, self-guided interventions require participants to be highly motivated and disciplined, which may not always be realistic, particularly for perinatal populations facing additional stressors.

Interventions also differed in the inclusion of multimedia components, such as videos, audio recordings and interactive exercises. While some studies [22, 43] included a mix of components aimed at increasing user engagement, others relied solely on text-based instructions. This variation in design complicates direct comparisons of intervention efficacy. As seen in other reviews of digital health interventions, such heterogeneity makes it difficult to determine which components are most effective in fostering psychological improvements [58].

Intervention duration and intensity also varied, with some programs lasting only a few weeks while others extended over several months. Shorter interventions may be more appealing to users with limited time, yet longer interventions could offer more sustained benefits. The current evidence does not provide a clear answer regarding the ideal length or intensity of mHealth MBIs for perinatal populations.

Given this variability, future research should aim to identify the most effective formats and intervention lengths. Comparative studies examining self-guided versus therapist-guided approaches, as well as exploring the role of non-expert guidance, could help to clarify these issues. Non-expert guided interventions, where community members are trained to facilitate mindfulness practices, might offer a scalable solution to improve access to mental health resources during the perinatal period [59].

### Practical implications for clinicians and app developers

The findings of this review emphasize the need for clinicians and app developers to collaborate in designing mHealth MBIs that address both user preferences and practical barriers. For clinicians, incorporating mHealth solutions into perinatal care could enhance accessibility to psychological support, particularly for individuals in underserved or rural areas. Clinicians should provide guidance on integrating these apps into daily routines, ensuring that participants understand their utility in managing stress, anxiety and depression.

For app developers, creating user-friendly designs that offer customizable features such as flexible session durations, reminders and progress tracking is crucial. Addressing common technical issues, such as compatibility across devices, and including culturally tailored content can further improve usability and engagement. Additionally, developers should consider incorporating structured follow-ups and feedback mechanisms to reduce dropout rates and enhance retention. Together, these strategies can help maximize the effectiveness and scalability of mHealth MBIs for perinatal psychological health.

### Strengths and limitations of the current study

This systematic narrative review provides valuable insights into the effectiveness of mHealth MBIs on perinatal psychological health. One of its key strengths is the inclusion of studies from diverse geographic locations and varying designs, such as RCTs and non-RCTs, which enhances the review’s comprehensiveness. The review also sheds light on the effectiveness of mHealth MBIs in both preventing and reducing symptoms of depression, anxiety and stress, aligning with broader evidence on the utility of digital mindfulness programs [23].

However, several limitations must be acknowledged. First, the study's reliance on a narrative synthesis, due to the heterogeneity of the included studies, precluded a meta-analysis that could have provided more robust quantitative insights. The variability in study design, sample sizes and intervention lengths further limits the ability to generalize findings across populations. Additionally, the review focuses predominantly on high-income countries, particularly the United States, which may not reflect the effectiveness of mHealth MBIs in low- and middle-income settings. None of the studies included in this review specifically examined the effectiveness of mHealth MBIs on individual pregnancy-related complications, such as GDM or PIH, nor did they explore the outcomes of these complications after the intervention.

Future research should address this gap by evaluating the applicability of these interventions in more diverse socio-economic and cultural contexts and by focusing on pregnancy-related complications to better understand the impact of mHealth MBIs on these specific circumstances.

## CONCLUSION

This review gathered the most recent evidence on the effectiveness of mHealth MBIs for perinatal psychological health. The findings suggest that while mHealth MBIs show promise in reducing symptoms of depression, anxiety and stress, the field remains in its early stages and substantial gaps in evidence persist. Future studies should prioritize rigorous methodologies, including RCTs with adequate sample sizes, to establish the clinical significance of observed improvements. Furthermore, research should focus on identifying optimal intervention formats, such as self-guided versus guided approaches, and strategies to enhance adherence through personalized features and culturally tailored content.

Long-term impact assessments are crucial to understanding the sustainability of benefits over time, particularly as the perinatal period presents unique psychological and practical challenges. Additionally, there is an urgent need to evaluate these interventions in diverse populations, including those in low-resource settings, to ensure global applicability. Addressing pregnancy-related complications such as GDM and pre-eclampsia through targeted interventions could also provide valuable insights into the broader utility of mHealth mindfulness programs.

Ultimately, advancing this field will require interdisciplinary collaboration among researchers, clinicians and app developers to design, implement and evaluate interventions that are not only evidence-based but also scalable and adaptable to the needs of perinatal populations worldwide.

## Supplementary Material

Appendix-OODH-PKGODAGE_oqaf006

## Data Availability

The data underlying this article will be shared on reasonable request to the corresponding author.
