# Supplementary material for: The effectiveness of mHealth mindfulness interventions on perinatal psychological health: a systematic review
Source: Oxf Open Digit Health. 2025 Mar 20;3:oqaf006. doi: 10.1093/oodh/oqaf006 (PMC11994998; doi:10.1093/oodh/oqaf006)
Supplement: Appendix-OODH-PKGODAGE_oqaf006 [file appendix-oodh-pkgodage_oqaf006.docx]

**Supplementary Materials Heading**

Appendix 1

**Manuscript title**

The Effectiveness of mHealth Mindfulness Interventions on Perinatal Psychological Health: A Systematic Review

**Authors**

Prabhadini Godage^1*^, Oonagh M. Giggins^1^, Julie Doyle^1^, Anita Byrne^2^

Affiliations

1. NetwellCASALA, School of Health and Science, Dundalk Institute of Technology, Dundalk, County Louth, Ireland

2. School of Health and Science, Dundalk Institute of Technology, Dundalk, County Louth, Ireland

Corresponding author: Prabhadini Godage

Email: Prabhadini.Godage@dkit.ie

Mailing address: Dundalk Institute of Technology, Dublin Road, Dundalk, County Louth, Ireland, A91 K584

**Appendix 1**

| **Database** | **Search Strategy** |
| --- | --- |
| PubMed | (Mobile Applications[Mesh] OR mobile app[All Fields] OR digital[All Fields] OR electronic[All Fields] OR online[All Fields] OR smartphone[All Fields] OR mHealth[All Fields] OR m-health[All Fields] OR health apps[All Fields] OR mobile health apps[All Fields] OR mHealth technologies[All Fields] OR healthcare mobile applications[All Fields] OR mobile-delivered[All Fields]) AND (Mindfulness[Mesh] OR mindful*[All Fields] OR mindful meditation[All Fields] OR mindfulness-Based stress reduction[All Fields] OR MBSR[All Fields] OR mindful awareness[All Fields] OR mind-body practices[All Fields] OR mindful therapy[All Fields] OR mindfulness training[All Fields] OR mindfulness-based cognitive therapy[All Fields] OR mindfulness-integrated cognitive behavioural therapy[All Fields] OR MBCT[All Fields] OR mindfulness-based intervention[All Fields] ) OR meditation[All Fields] AND (mental health[Mesh] OR stress[All Fields] OR psychological stress[All Fields] OR mental stress[All Fields] OR emotional strain[All Fields] OR stressor*[All Fields] OR stress management[All Fields] OR coping with stress[All Fields] OR stress reduction[All Fields] OR anxiety[All Fields] OR depression[All Fields] OR fear[All Fields]) AND (Pregnancy[Mesh] OR pregnan*[All Fields] OR perinatal[All Fields] OR perinatal period[All Fields] OR prenatal period[All Fields] OR postnatal[All Fields] OR maternal health[All Fields] OR antenatal care[All Fields] OR pregnancy outcome[All Fields] OR gestation[All Fields] OR obstetrics[All Fields] OR complex pregnancy[All Fields] OR pregnancy complication[All Fields] OR GDM[All Fields] OR gestational diabetes mellitus[All Fields] OR PIH[All Fields] OR pregnancy induced hypertension[All Fields] OR heart disease in pregnancy[All Fields]) |
| Cochrane Library | #1 MeSH descriptor: [Mobile Applications] explode all trees  #2 (Mobile Applications OR mobile app OR smartphone OR digital OR electronic OR online OR mHealth OR m-health OR health apps OR mobile health apps OR mHealth technologies OR healthcare mobile applications OR mobile-delivered)  #3 MeSH descriptor: [Mindfulness] explode all trees  #4 (Mindfulness OR mindful OR mindful meditation OR mindfulness-Based stress reduction OR MBSR OR mindful awareness OR mind-body practices OR mindful therapy OR mindfulness training OR mindfulness-based cognitive therapy OR mindfulness-integrated cognitive behavioral therapy OR MBCT OR mindfulness-based intervention)  #5 MeSH descriptor: [Mental Health] explode all trees  #6 (mental health OR stress OR psychological stress OR mental stress OR emotional strain OR stressor OR stress management OR coping with stress OR stress reduction OR anxiety OR depression OR fear)  #7 MeSH descriptor: [Pregnancy] explode all trees  #8 (Pregnancy OR pregnant OR perinatal OR perinatal period OR prenatal period OR postnatal OR maternal health OR antenatal care OR pregnancy outcome OR gestation OR obstetrics OR complex pregnancy OR pregnancy complication OR GDM OR gestational diabetes mellitus OR PIH OR pregnancy induced hypertension OR heart disease in pregnancy)  #9 (#1 OR #2) AND (#3 OR #4) AND (#5 OR #6) AND (#7 OR #8) |
| Science Direct | (Mobile applications OR mHealth) AND (Mindfulness OR Mindfulness-Based Stress Reduction) AND (Mental Health OR Stress) AND (Pregnancy OR Perinatal) |
| Scopus | (TITLE-ABS-KEY(mobile applications) OR TITLE-ABS-KEY(mobile phone) OR TITLE-ABS-KEY(mhealth) OR TITLE-ABS-KEY(mhealth apps) OR TITLE-ABS-KEY(mhealth app) OR TITLE-ABS-KEY(smartphone) OR TITLE-ABS-KEY(digital) OR TITLE-ABS-KEY(electronic) OR TITLE-ABS-KEY(online) AND TITLE-ABS-KEY(mindfulness) OR TITLE-ABS-KEY(mindfulness based stress reduction) OR TITLE-ABS-KEY(mindfulness meditation) OR TITLE-ABS-KEY(mindfulness based cognitive therapy) AND TITLE-ABS-KEY(mental health) OR TITLE-ABS-KEY(stress response) OR TITLE-ABS-KEY(psychological stress) OR TITLE-ABS-KEY(stressors) OR TITLE-ABS-KEY(stressor) OR TITLE-ABS-KEY(anxiety) OR TITLE-ABS-KEY(depression) OR TITLE-ABS-KEY(fear) AND TITLE-ABS-KEY(pregnancy) OR TITLE-ABS-KEY(pregnant women) OR TITLE-ABS-KEY(pregnancy complications) OR TITLE-ABS-KEY(perinatal) OR TITLE-ABS-KEY(prenatal) OR TITLE-ABS-KEY(maternal health) OR TITLE-ABS-KEY(antenatal) OR TITLE-ABS-KEY(antenatal care) OR TITLE-ABS-KEY(gestation) OR TITLE-ABS-KEY(obstetrics) OR TITLE-ABS-KEY(obstetric) OR TITLE-ABS-KEY(postnatal) OR TITLE-ABS-KEY(gdm) OR TITLE-ABS-KEY(gestational diabetes mellitus) OR TITLE-ABS-KEY(pih)) |
| AMC Digital Library | [[All: "mobile applications"] OR [All: "mobile app"] OR [All: "smartphone"] OR [All: "digital"] OR [All: or]] AND [[All: "electronic" "online"] OR [All: "mhealth"] OR [All: "m-health"] OR [All: "health apps"] OR [All: "mobile health apps"] OR [All: "mhealth technologies"] OR [All: "healthcare mobile applications"] OR [All: "mobile-delivered"]] AND [[All: "mindfulness"] OR [All: "mindful meditation"] OR [All: "mindfulness-based stress reduction"] OR [All: "mindful awareness"] OR [All: "mind-body practices"] OR [All: "mindful therapy"] OR [All: "mindfulness training"] OR [All: "mindfulness-based cognitive therapy"] OR [All: "mindfulness-integrated cognitive behavioural therapy"] OR [All: "mindfulness-based intervention"]] AND [[All: "mental health"] OR [All: "stress"] OR [All: "psychological stress"] OR [All: "mental stress"] OR [All: "emotional strain"] OR [All: "stressors"] OR [All: "stress management"] OR [All: "coping with stress"] OR [All: "stress reduction"] OR [All: "anxiety"] OR [All: "depression"] OR [All: "fear"]] AND [[All: "pregnancy"] OR [All: "pregnant"] OR [All: "perinatal"] OR [All: "perinatal period"] OR [All: "prenatal period"] OR [All: "maternal health"] OR [All: "antenatal care"] OR [All: "pregnancy outcome"] OR [All: "gestation"] OR [All: "obstetrics"] OR [All: "postnatal"] OR [All: "complex pregnancy"] OR [All: "pregnancy complication"] OR [All: "gdm"] OR [All: "gestational diabetes mellitus"] OR [All: "pih"] OR [All: "pregnancy induced hypertension"]] |
| IEEE Xplore | ("Full Text & Metadata":"mobile app*" OR "Full Text & Metadata":mhealth* OR "Full Text & Metadata": "ehealth technologies") AND ("Full Text & Metadata":mindful* OR "Full Text & Metadata":"mindful meditation" OR "Full Text & Metadata":"mindfulness based stress reduction") AND ("Full Text & Metadata":"mental Health" OR "Full Text & Metadata":stress* OR "Full Text & Metadata":"anxiety" OR "Full Text & Metadata":"depression") AND ("All Metadata":pregnancy* OR "All Metadata":perinatal* OR "All Metadata":prenatal* OR "All Metadata":maternal* OR "All Metadata": gestation OR "All Metadata":obstetrics OR "All Metadata":gestational diabetes mellitus OR "All Metadata":pregnancy induced hypertension OR "All Metadata":heart disease in pregnancy) |
